# Ocean Acidification Affects the Phyto-Zoo Plankton Trophic Transfer Efficiency

**DOI:** 10.1371/journal.pone.0151739

**Published:** 2016-04-15

**Authors:** Gemma Cripps, Kevin J. Flynn, Penelope K. Lindeque

**Affiliations:** 1 Ocean and Earth Sciences, University of Southampton, National Oceanography Centre, Southampton, United Kingdom; 2 Biosciences, Swansea University, Swansea, United Kingdom; 3 Marine Ecology and Biodiversity, Plymouth Marine Laboratory, Plymouth, United Kingdom; University of Connecticut, UNITED STATES

## Abstract

The critical role played by copepods in ocean ecology and biogeochemistry warrants an understanding of how these animals may respond to ocean acidification (OA). Whilst an appreciation of the potential direct effects of OA, due to elevated *p*CO_2_, on copepods is improving, little is known about the indirect impacts acting via bottom-up (food quality) effects. We assessed, for the first time, the chronic effects of direct and/or indirect exposures to elevated *p*CO_2_ on the behaviour, vital rates, chemical and biochemical stoichiometry of the calanoid copepod *Acartia tonsa*. Bottom-up effects of elevated *p*CO_2_ caused species-specific biochemical changes to the phytoplanktonic feed, which adversely affected copepod population structure and decreased recruitment by 30%. The direct impact of elevated *p*CO_2_ caused gender-specific respiratory responses in *A*.*tonsa* adults, stimulating an enhanced respiration rate in males (> 2-fold), and a suppressed respiratory response in females when coupled with indirect elevated *p*CO_2_ exposures. Under the combined indirect+direct exposure, carbon trophic transfer efficiency from phytoplankton-to-zooplankton declined to < 50% of control populations, with a commensurate decrease in recruitment. For the first time an explicit role was demonstrated for biochemical stoichiometry in shaping copepod trophic dynamics. The altered biochemical composition of the CO_2_-exposed prey affected the biochemical stoichiometry of the copepods, which could have ramifications for production of higher tropic levels, notably fisheries. Our work indicates that the control of phytoplankton and the support of higher trophic levels involving copepods have clear potential to be adversely affected under future OA scenarios.

## 1.Introduction

Mesozooplankton play a crucial role within marine food webs, transferring biomass from primary producers to higher trophic levels, and in doing so significantly contributing to the vertical particle flux. As copepods typically form a significant proportion of the mesozooplankton [1], any influence on their survival, growth or development attributed to ocean acidification (OA) may be expected to have significant implications on trophic dynamics.

To gain an understanding of the potential impacts of OA upon marine organisms, experiments are typically conducted under elevated partial pressures of carbon dioxide (*p*CO_2_), ideally using *p*CO_2_ values consistent with predicted future atmospheric CO_2_ concentrations. In copepods, the direct effects of elevated *p*CO_2_ have shown to vary between species [2], populations [3], and developmental stages within a species [4,5,6]. The extent of these direct effects appears to be related to the duration of exposure to OA, with recent transgenerational studies demonstrating diminishing effects with prolonged exposure [7]. While our understanding of the direct effects of elevated *p*CO_2_ on copepods is improving [7,8], little is known of the indirect impacts that OA may cause on copepod populations through indirect, bottom-up, effects mediated through effects of OA on copepod prey [9]. The increase in CO_2(aq)_ in the water column, associated with OA, is suspected to have the potential to increase the carbon-nutrient (e.g., C:N, C:P) ratios of primary producers [10,11,12]. If this was indeed to occur, then the consequential changes in the elemental stoichiometry of the primary producers could translate to poor-quality prey for consumers with decreased trophic transfer efficiency [13] that affects biogeochemistry. Growth under elevated *p*CO_2_ also has the potential to alter the biochemical composition of primary produces [14,15,16]. Changes in biochemical content can affect the consumer’s reproduction and development through insufficient supply of critical metabolites [17,11], and thus change the efficiency of energy transfer between the producer and consumer.

In addition to the above mentioned interactions of OA upon trophic transfer, behavioural interactions between predator and prey across marine taxa have also shown to be affected by the projected changes in seawater carbonate chemistry associated with OA [18,19,20]. Although the mode of action remains unclear, copepods have an ability to discriminate between prey types based on size [21] and motility [22], as well as the presence of noxious substances produced by prey [23]. Indeed, copepods have the potential to actively select higher quality prey species with lower C:(N:P) ratios [24,25], when the nutritional variance within the prey is notable [26].

Taken all together, there is scope for OA to affect copepod growth and reproduction and thence interactions to trophic levels below them (their phytoplankton prey) and above (through to fisheries), and associated biogeochemical cycles. A primary driver may be expected to depend on the response of the prey to OA, the number of prey types and quantities available, and if appropriate the predator’s ability to detect the changes in prey quality and choose an alternative prey source.

In this study, we explored the direct (via increased external *p*CO_2_ seawater), indirect (via mixed-prey [*Isochrysis galbana*, *Tetraselmis suecica* and *Chaetoceros muelleri*] reared under increased *p*CO_2_) and combined (simultaneous direct and indirect exposure) effects of OA on the ubiquitous calanoid copepod *Acartia tonsa*. To assess if the combined exposure caused a multiplicative effect on the consumer, a cross factorial design of predator and prey reared under elevated (1000 μatm) and low (ambient: 400 μatm) *p*CO_2_ levels was utilised to locate sole stressor effects. Vital rates (ingestion, respiration rates and reproduction), behaviour (prey selection) and composition (elemental and biochemical stoichiometry) were measured in copepods after being exposed to *p*CO_2_ levels in-line with near-future OA scenarios for one life-cycle. Implications of the different OA pathways on the trophic interactions between phytoplankton and zooplankton were subsequently calculated through elemental and biochemical stoichiometric trophic transfer efficiencies.

## 2.Method

### 2.1. Carbonate chemistry

The calanoid copepod *Acartia tonsa* and its phytoplanktonic prey (prymnesiophyte *Isochrysis galbana* [CCAP 927/ 1], prasinophyte *Tetraselmis suecica* [CCAP 66/ 22C] and diatom *Chaetoceros muelleri* [CCAP 927/ 1]) were separately grown under two *p*CO_2_ scenarios; (i) low: present-day *p*CO_2_ concentrations of 400 μatm, and (ii) elevated: worst-case scenario for the year 2100, 1000 μatm (RCP 8.5 [27]). The details of the method used to achieve these scenarios is outlined in S1 Text, and absolute concentrations for each nominal treatment is detailed in S2 Table. These two *p*CO_2_ concentrations were combined in a matrix between the two trophic levels to produce 4 treatments: (i) Z_L_P_L_: zooplankton (*A*. *tonsa*) reared under low *p*CO_2_ levels fed mixed phytoplankton (I. *galbana*, *C*. *muelleri and T*. *suecica*) also reared under low *p*CO_2_ levels, (ii) Z_L_P_E_: zooplankton reared under low *p*CO_2_ levels fed mixed phytoplankton reared under elevated (RCP 8.5) *p*CO_2_ levels, (iii) Z_E_P_L_: zooplankton reared under elevated *p*CO_2_ levels fed mixed phytoplankton reared under low *p*CO_2_ levels, (iv) Z_E_P_E_: zooplankton reared under elevated *p*CO_2_ levels fed mixed phytoplankton also reared under elevated *p*CO_2_ level.

### 2.2. Experimental design

#### Phytoplankton

Phytoplankton prey species were cultured separately in nutrient replete seawater-based medium (88.2 and 5.5 μmol L^-1^ for NO_3_^-^ and PO_4_^3-^ respectively; mole N: P ratio 16: 1) in semi-continuous cycles (effective dilution rate: *T*. *suecica* 0.30 d^-1^, *I*. *galbana* and *C*. *muelleri*: 0.35 d^-1^) for a minimum of 12 generations. Cultures were grown in a 18:6 hour light: dark cycle (cool-white fluorescent tubes at 50 μmol photons m^-2^ s^-1^) at 22 ± 1.8°C. Duplicate cultures of each species were used for both *p*CO_2_ treatments (450 mL, total n = 18). *I*. *galbana*, *C*. *muelleri* and *T*. *suecica* cultures (500 mL flasks) were aerated with air at the required *p*CO_2_ concentration (either low or elevated) through a sterilised glass airline via an air-filter (0.2μm) at a flow rate of ca. 52 mL min^-1^. Cell number (cells mL^-1^), size (as equivalent spherical diameter, μm) and biovolume (μm^3^ mL^-1^) for all replicates were analysed at the end of each light cycle using a Multisizer 4 Coulter Counter (Beckman, USA). Every 48hrs, at the semi-continuous exchange point, cells were collected from each culture for elemental stoichiometry and biochemical analysis. Cellular carbon (μg C mL^-1^), nitrogen (μg N mL^-1^) and the C: N of each species grown under both *p*CO_2_ concentrations were analysed using an elemental analyser coupled with an isotope ratio mass spectrometer (SerCon GSL). Relative biochemical stoichiometry (lipids: protein, protein: carbohydrate, and carbohydrate: lipid) of each species cultured at different *p*CO_2_ concentrations was assessed through Fourier Transform Infra-red (FTIR) spectroscopy (PerkinElmer Spectrum 2), over a wavelength range of 450–4000 cm^-1^ and at a resolution of 4 cm^-1^. The methods employed for the FTIR measurements are described in Mayers *et al* [28], and the quantification of relative biochemical stoichiometry in Stehfast *et al* [29], and as described further in S1 Text.

#### Copepod vital rates

Copepods were exposed to the four treatments for an entire life cycle, from generation 1(G_1_) early nauplii stages (N_I_) through to G_2_ mid-late nauplii stages (N_III-IV_). Each treatment had four replicate populations (1L), initiated with N_I_ at density 890 ind^-1^ L^-1^. Fecundity success, respiration rates and ingestion rates of mature adults were measured across the four treatments after a complete life cycle of exposure to the *p*CO_2_ conditions. For fecundity success, 5- 8 females from each replicate population (n = 20- 32 individuals per treatment) with an attached spermatophore were removed and placed individually into 30 mL vials filled with medium of their assigned treatment and with saturating prey quantities of their allocated mixed-prey (>1μg C mL^-1^). Each vial was pre-lined with a 150 μm nylon mesh bottom to separate eggs from the female to prevent egg cannibalism. Females were held for 24–30 hours to lay eggs. Egg production rates (EPR; eggs female^-1^ day^-1^), egg hatching success (EHS [%]) and nauplii recruitment (NR; nauplii female^-1^ day^-1^) across the four treatments were calculated as described in Cripps *et al* [6]. Ingestion rates (μg C ind^-1^ day^-1^) of adult males and females were measured separately. A sufficient number of adult copepods (males: 250 ind^-1^ L^-1^, females:170 ind^-1^ L^-1^) were transferred from the experimental population replicates to 60 mL tissue culture flasks (6–8 replicates per life stage, male and female, for each treatment) filled with filtered (0.2 μm) sterilised seawater of the required *p*CO_2_ concentration. Prey (*I*. *galbana*, *C*. *muelleri* and *T*. *suecica*), reared under low or elevated *p*CO_2_, were then added to the corresponding predator tissue culture flasks at the same concentration as used for the stock populations. After 24 hours, ingestion rates were calculated across the 4 treatments using Frost’s [21] equations. Respiration rates (nL O_2_ ind^-1^ min^-1^) were calculated over a period of 6–8 hours separately for adult males and females (8–10 replicates per life stage per treatment) using a non-invasive optical fluorescence-based oxygen respirometry (Fibox 3 LCD trace transmitter, PreSens, Germany). The method employed is detailed further in S1 Text.

#### Copepod prey selectivity

Adult male and female prey preference under direct, indirect and combined exposure to elevated *p*CO_2_, were calculated from the ingestion rates using Chesson’s prey selection index [30].

#### Elemental and biochemical stoichiometry of copepods

Mature males and females (between 1–5 days old) were collected for elemental stoichiometry (μg C ind^-1^, μg N ind^-1^ and C: N) and biochemical stoichiometry (lipid: protein, protein: carbohydrate, and carbohydrate: lipid) across the four treatments. The carbon and nitrogen content of the adults were measured separately for males (8–10 replicates per treatment, 15–25 individuals per replicate) and females (8–10 replicates per treatment, 10–15 individuals per replicate). Individuals were placed into tin cups (6x4 mm; Exeter Analytical, UK), immediately frozen and stored at -80°C until analysis. The relative difference between the biochemical compositions of *A*. *tonsa* adults were assessed using FTIR analysis. Individuals were pipetted into 1.5 mL micro-centrifuge tubes, frozen at -80°C, freeze dried (< 24 hours after initial freezing) and then homogenised prior to FTIR analysis. For both elemental and biochemical analyses the same methods were used as described for prey.

#### Trophic transfer

The influence of different *p*CO_2_ treatments (direct and/or indirect) on the trophic transfer efficiency was calculated using the carbon allocation budgets of adult females in G_1_. All measured metabolic rates were converted into carbon equivalents; ingestion rates (I, gC gC^-1^ d^-1^), EPR were used as an index for female growth (G, gC gC^-1^ d^-1^), and respiration rates (nL O_2_ ind^-1^ min^-1^) were converted into respiratory carbon equivalents (R, gC gC^-1^ d^-1^) using the respiratory quotient of 0.97 [31,32]. The proportion of carbon ingested (I) that was allocated to growth (G) was calculated as Gross Growth Efficiency (GGE = G/I). The proportion of carbon incorporated into growth in relation to the total carbon assimilated was calculated as Net Growth Efficiency (NGE = G/ G+R). The standard deviation (Xσ) for the calculated transfer efficiencies (NGE and GGE) and weights-specific rates (I, R and G) were calculated to incorporate error propagation. Correlations between the biochemical stoichiometric multivariate responses of the prey (lipid: protein, lipid: carbohydrate and protein: carbohydrate of *C*. *muelleri*, *I*. *galbana* and *T*. *suecica* under both P_L_ and P_E_) to that of the predator (lipid: protein, lipid: carbohydrate and protein: carbohydrate of Z_L_P_L_, Z_E_P_L_, Z_L_P_E_ and Z_E_P_E_ populations) were analysed using a Mantel test. Multiple stepwise search analyses were used to determine which biochemical ratio from the prey best matched the multivariate pattern of the predator’s biochemical stoichiometric composition, using the BVSTEP routine.

### 2.3. Statistical analyses

#### Phytoplankton

The influence of *p*CO_2_ on the growth rates (cells mL^-1^ and BV μm^3^ mL^-1^), cell size (μm), carbon content (μg C), nitrogen content (μg N) and C:N ratios of the three phytoplankton species were analysed using permutational multivariate analysis of variance (PERMANOVA). All dependent variables were assembled into a resemblance matrix using Euclidean distance and analysed using a factorial design with two crossed fixed factors; (i) species (*I*. *galbana*, *T*. *suecica* and *C*. *muelleri*), and (ii) treatment (P_L_ and P_E_). An additional nested factor of time was incorporated into the ‘treatment’ factor for two of the dependent variables (growth rate and cell size). Main effects and pairwise comparisons of the different factors were analysed through unrestricted permutations of raw data. If a low number of permutations were generated then the *p*-value was obtained through random sampling of the asymptotic permutation distribution, using Monte Carlo tests. For each dependent variable the dispersion across the factors was first analysed using permutational dispersion. Because cell size had a significantly different dispersion across the different *p*CO_2_ levels (both, *p* = < 0.05), cell size was transformed (log (χ+ 1)) prior to the PERMANOVA analysis. Fixed factor (P_L_ and P_E_) multivariate analysis (PERMANOVA) was used to compared the combined biochemical stoichiometry between the treatments for each species, followed by a one-way fixed factor analysis of variance to compare each stoichiometric ratio between the 2 *p*CO_2_ treatments (P_L_ and P_E_). The lipid: carbohydrate, lipid: protein and carbohydrate: protein ratios in *I*. *galbana* were transformed prior to analysis, as each ratio had a significantly different dispersion across the different *p*CO_2_ levels (*p* = < 0.05). An α-level of *p* = ≤ 0.05 was used for assessing statistical significance. Analyses were carried out in PRIMER-e (version 6.1.15) with the PERMANOVA add-on (version 1.0.3, Plymouth Marine Laboratory, Plymouth, UK) and R-software (version 3.2.1).

#### Copepods

The influence of direct, indirect and combined elevated *p*CO_2_ exposure on the individual vital rates (fecundity success [EPR: female^-1^ day^-1^, ES: μm^3^, EHS: % and NR: female^-1^ day^-1^], ingestion rates [μg C ind^-1^ day^-1^] and respiration rates [nL O_2_ ind^-1^ min^-1^]), behaviour (ɑ-index) and elemental stoichiometry (C, N and C:N) of *Acartia tonsa* were analysed using PERMANOVA factorial design with two crossed fixed factors; (i) treatment (Z_L_P_L_, Z_E_P_L_, Z_L_P_E_ and Z_E_P_E_) and (ii) life stage (for respiration and ingestion only). Differences in the copepods relative biochemical compositions between the treatments were analysed using the same method employed for the phytoplankton. Means and calculated standard deviations of trophic transfer efficiencies (GGE and NGE) and weights-specific rates (I, R and G) were compared through a fixed-factor analysis of variance design between the treatments. Correlations between the multivariate biochemical stoichiometric ratios of the prey and the predators were assessed through a Mantel test, using Spearman’s rank correlation coefficient (rho). Multiple stepwise search analyses (BVSTEP) determined which biochemical component across the 3 prey species (lipid: protein, lipid: carbohydrate and protein: carbohydrate of *C*. *muelleri*, *I*. *galbana* and *T*. *suecica* under both P_L_ and P_E_) had the greatest influence on the predator’s composition (lipid: protein, lipid: carbohydrate and protein: carbohydrate of Z_L_P_L_, Z_E_P_L_, Z_L_P_E_ and Z_E_P_E_ populations). The BVSTEP routine successively adds and removes a variable to obtain the optimum correlation between the zooplankton and prey’s composition, using spearman’s correlation coefficient. An α-level of *p* = ≤ 0.05 was used for assessing statistical significance across main tests, and Bonferroni corrections were incorporated during multiple testing between the 4 treatments using an α-level of *p* = ≤ 0.0125.

## 3.Results

Throughout the following, subscript L and subscript E refer to treatments as low (ambient) or elevated (OA) *p*CO_2_ respectively, as applied to zooplankton (i.e., Z_L_, Z_E_) or phytoplankton (i.e., P_L_, P_E_). Direct treatments are thus indicated as Z_E_P_L_, indirect as Z_L_P_E_, and combined as Z_E_P_E_, with the control as Z_L_P_L_.

### 3.1. Phytoplankton

**Fig 1:**
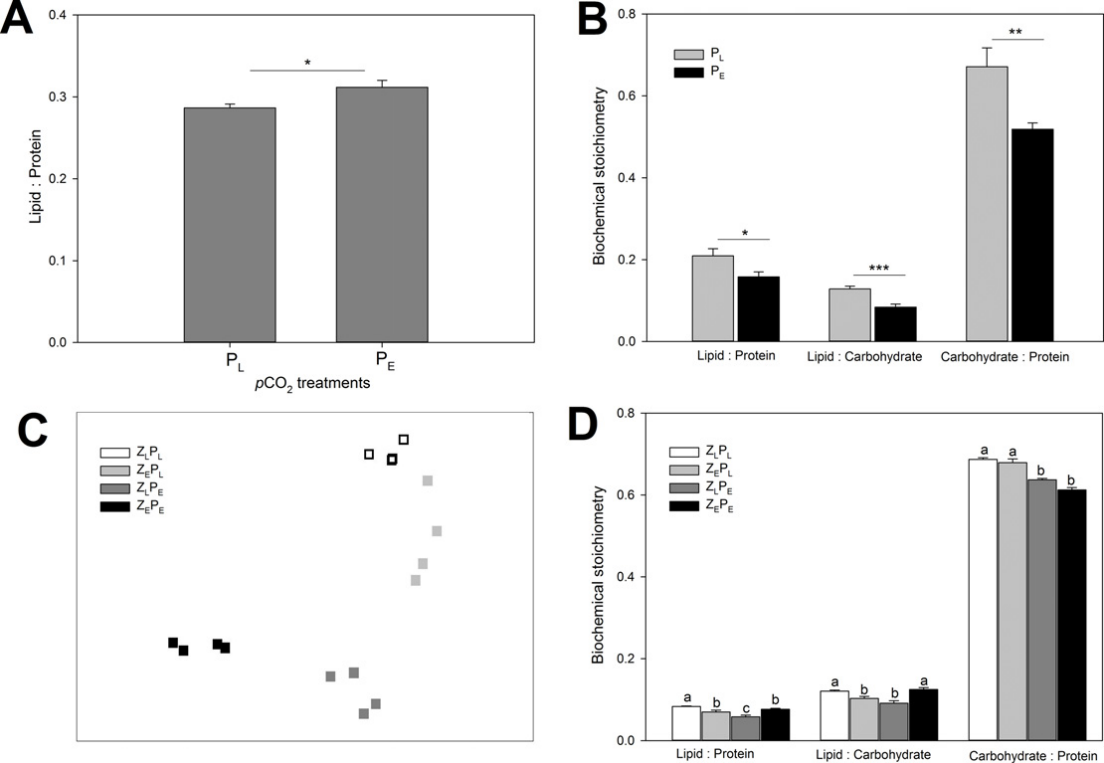
**Biochemical stoichiometry of phytoplankton prey (A, B) and adult predator copepods (C, D) upon exposure to 4 different OA treatments.** **A**: The lipid: protein ratio of *C*. *muelleri* reared at ambient (P_L_) and elevated (P_E_) *p*CO_2_ levels. **B:** The lipid: protein, lipid: carbohydrate and protein: carbohydrate ratio of *I*. *galbana* reared at ambient and elevated *p*CO_2_ levels. Stars denote significance differences between the 2 treatments: *** = *p* < 0.001, ** = *p* < 0.01 and * = *p* < 0.05. **C:** Multi-dimensional ordinal scale (nMDS) plot representing the ordinal distance between the biochemical stoichiometry of *A*. *tonsa* adult populations exposed to 4 different *p*CO_2_ treatments for one-life cycle (Z_L_P_L_ = both plankton prey and copepod predators reared under ambient *p*CO_2_ levels, Z_E_P_L_: prey reared under ambient *p*CO_2_ levels and predators reared under elevated levels, Z_L_P_E_: prey reared under elevated *p*CO_2_ levels and predators reared under ambient levels, and Z_E_P_E_: both prey and predator reared under elevated *p*CO_2_ levels). **D**: The variation in biochemical ratios across the four *p*CO_2_ treatments in adult *Acartia tonsa*. Letters denote significant difference between the 4 treatments within each group (biochemical ratio). Columns that do not share the same letter are significantly different from one another. The integrated band ratios assigned for each biochemical group are detailed in S1 Table. Corresponding *p*CO_2_ treatment concentrations are detailed in S2 Table. Values are average ± 1SE across all graphs.

No differences were found in the growth rates, cell size, or elemental content (carbon, nitrogen, C: N) across the 3 phytoplankton species tested as a result of growth at elevated *p*CO_2_. However, species-specific differences were found in the biochemical composition as determined by Fourier Transform Infrared Spectroscopy (FTIR). The biochemical stoichiometry of the diatom *C*. *muelleri* and prymnesiophyte *I*. *galbana* differed significantly under elevated *p*CO_2_ (multivariate analysis: *p* = 0.014, *F* = 6.25 and *p* = 0.002, *F* = 9.47, respectively) while no differences were found in the prasinophyte *T*. *suecica*. The lipid: protein ratio in *C*. *muelleri* was significantly higher under elevated *p*CO_2_ levels (1-way analysis of variance: *p* = 0.014, *F* = 6.25, Fig 1A). Variations between the lipid: carbohydrate ratios and the protein: carbohydrate ratios in *C*.*muelleri* could not be assessed, as the diatoms silicate peak obstructed the carbohydrate reading on the FTIR. For *I*. *galbana*, all biochemical stoichiometric ratios varied significantly between the two treatments, Fig 1B (1-way analysis of variance: lipid: protein: *p* = 0.017, F = 6.3749, lipid: carbohydrate: *p* = 0.001, *F =* 21.54 and protein: carbohydrate: *p* = 0.005, *F =* 8.65).

### 3.2. Copepods

#### Copepod chemical stoichiometry

Variations in carbon (μg C ind^-1^) and nitrogen (μg N ind^-1^) between the 4 treatments were found in adult males (multivariate analysis of variance: *p* = 0.001, *F* = 7.912), but not in adult females. Both carbon (C) and nitrogen (N) content in males increased in populations exposed to the combined elevated *p*CO_2_ conditions (pairwise-test: C: *p* = 0.007, *t* = 4.46 and N: *p* = 0.001, *t* = 7.12), though the C: N ratios in males were not found to be different between the 4 treatments. The biochemical composition of copepods varied across the treatments (multivariate analysis of variance: *p =* 0.001, *F* = 92.62), with the greatest stoichiometric similarities found between low *p*CO_2_ controls and direct OA treatments, where only the copepods were exposed to elevated *p*CO_2_ (Z_L_P_L_ vs Z_E_P_L_ in Fig 1C). All biochemical stoichiometric ratios significantly differed between the 4 treatments (1-way analysis of variance, lipid: protein: *p =* 0.001, *F* = 50.24, carbohydrate: protein: *p =* 0.002, *F* = 142.88, and lipid: carbohydrate: *p =* 0.001, *F* = 27.48). Copepod carbohydrate: protein ratios were significantly higher in control populations compared to populations exposed to the indirect and combined OA treatment (pairwise-test: Z_L_P_E_: *p* = 0.001, *t* = 19.28, and Z_E_P_E_: *p* = 0.001, *t* = 21.76, Fig 1D). The lipid: carbohydrate ratios significantly declined across the indirect and direct pathways (pairwise-test: Z_L_P_E_: *p* = 0.001, *t* = 9.00, and Z_E_P_L_: *p* = 0.004, *t* = 6.29, Fig 1D), but not in the combined OA treatment. The lipid: protein ratios of the copepods declined across all treatments compared to the ambient populations (pairwise-test: *p* < 0.0125 across all treatment ratios [Fig 1D]), with the greatest declines found across the individual *p*CO_2_ pathways (direct or indirect pathways).

#### Prey selectivity

**Fig 2:**
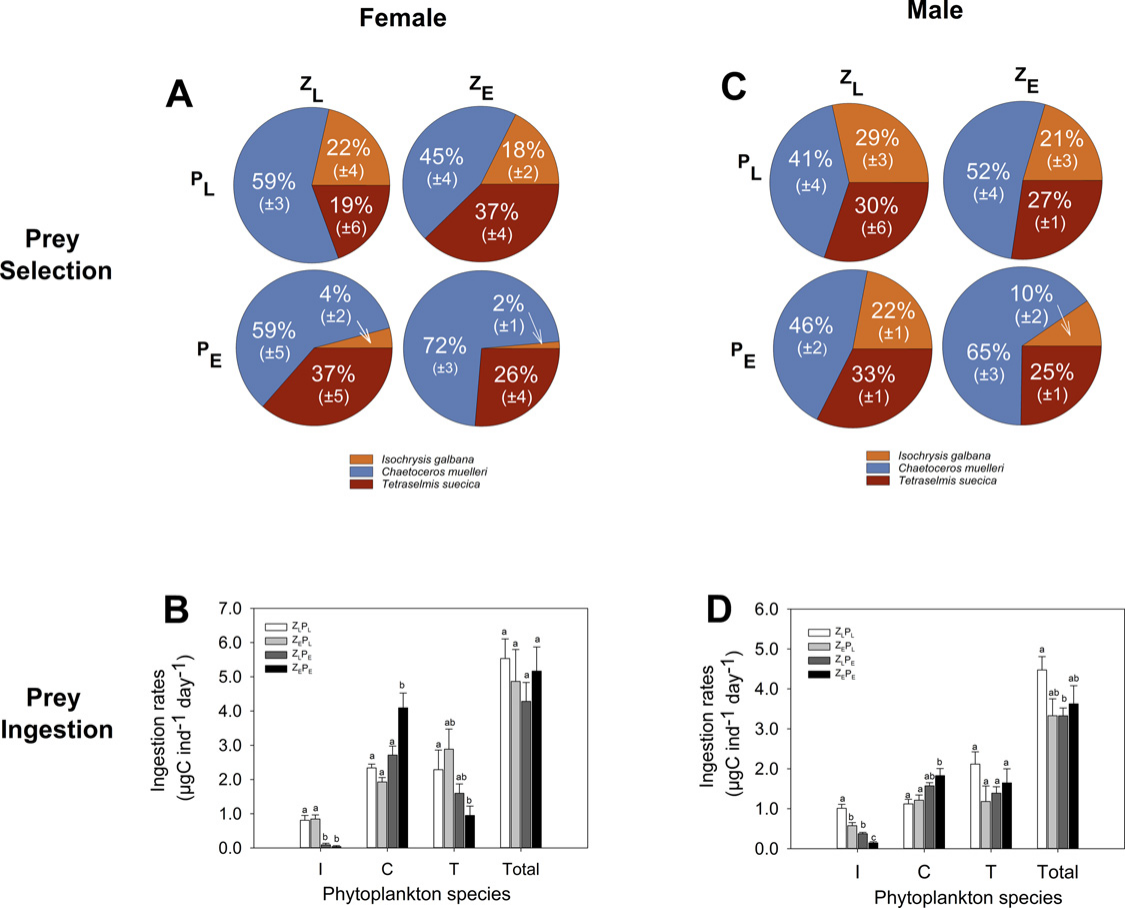
Prey selection and ingestion rates of adult *Acartia tonsa* exposed to 4 different OA treatments for one-life cycle. **A, C**: Prey selectivity (% of α-index) of adult females and males (respectively). **B, D:** Female and male ingestion rates of *I*. *galbana* (I), *C*. *muelleri* (C) and *T*. *suecica* (T). Letters denote significant difference between the 4 treatments within each group (i.e., male and female). Columns that do not share the same letter are significantly different from one another. Corresponding *p*CO_2_ treatment concentrations are detailed in S2 Table.

*C*. *muelleri* was preferentially selected by adult copepods across all 4 treatments (Fig 2A and 2B). The index (α-level) of prey selectivity for *C*. *muelleri* was significantly greater in females exposed to the combined elevated *p*CO_2_ treatment (>70%) compared to females preying on phytoplankton reared in ambient *p*CO_2_ levels (pairwise- test, Z_L_P_L_: *p =* 0.012, *t =* 3.63 and Z_E_P_L_: *p* = 0.003, *t* = 5.50 [Fig 2A]). This was also found in male populations, with individuals actively selecting *C*. *muelleri* to a greater extent (> 65%) under the combined elevated *p*CO_2_ treatment compared to ambient conditions (pairwise- test: *p =* 0.002, *t =* 4.16 [Fig 2B]).

#### Prey ingestion

While the total amount of prey (in terms of phytoplankton-carbon) ingested by adult females did not vary significantly with *p*CO_2_ exposure, females across the 4 different treatments attained this same total ingestion rate by consuming different prey types (Fig 2A and 2C). The combined direct and indirect exposure to elevated *p*CO_2_ led to a significantly greater consumption of *C*. *muelleri* (pairwise- test: *p =* 0.003, *t* = 4.87) with lowered ingestion rates of *I*. *galbana* (pairwise- test, *p* = 0.004, *t* = 4.19). For adult males, the overall ingestion rate varied between the treatments (1-way analysis of variance: *p* = 0.045, *F* = 3.06), and was significantly lower in populations that were exposed to indirect elevated *p*CO_2_ levels (pairwise- test: *p =* 0.008, *t =* 2.96). Similar to females, males also ingested *C*. *muelleri* at a greater rate under the combined elevated *p*CO_2_ treatment (pairwise- test, *p* = 0.005, *t* = 3.49, Fig 2B and 2D).

#### Respiration

**Fig 3:**
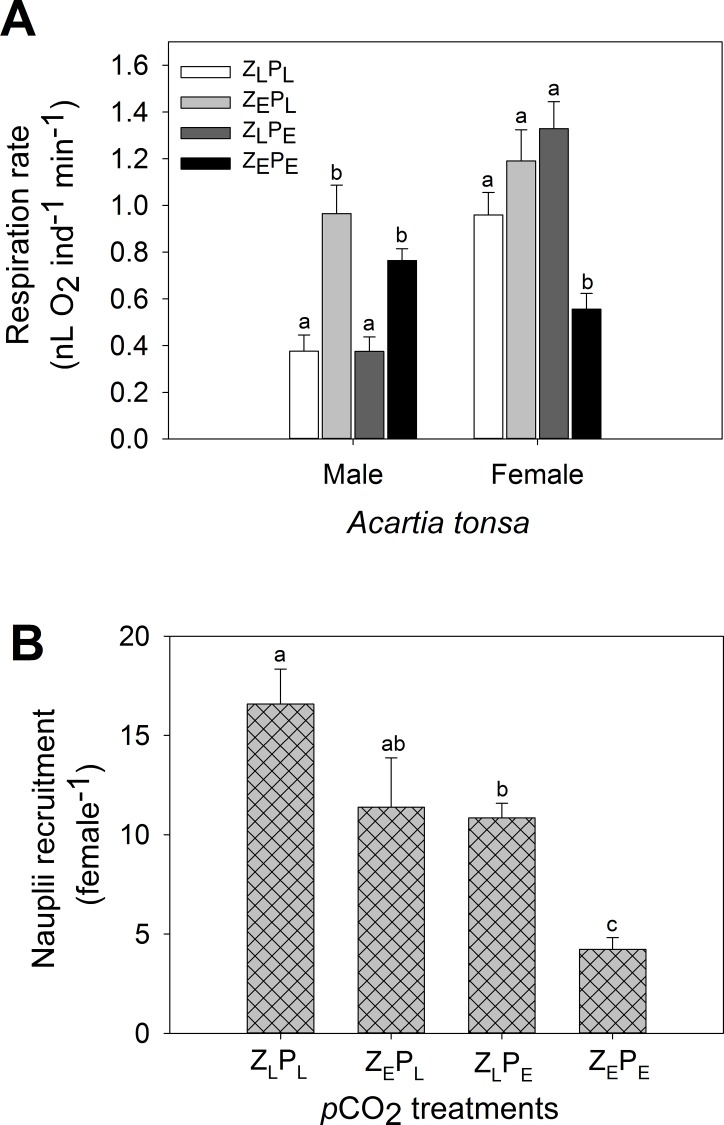
Vital rates of *Acartia tonsa* exposed to 4 different OA treatments after one-life cycle of exposure. **A**: Respiration rates of adult males and females, **B**: Nauplii recruitment per adult female. Letters denote significant difference between the 4 treatments within each group (i.e., male and female). Columns that do not share the same letter are significantly different from one another. Corresponding *p*CO_2_ treatment concentrations are detailed in S2 Table.

Respiration rates varied significantly across the 4 treatments in both adult males (1-way analysis of variance: *p* = 0.002, *F* = 9.04), and adult females (1-way analysis of variance: *p* = 0.003, *F* = 7.86, Fig 3A). Adult males directly exposed to elevated *p*CO_2_ levels (Z_E_P_E_ and Z_E_P_L_) displayed respiration rates 2–2.5 fold higher than males directly exposed to ambient *p*CO_2_ levels (pairwise- test: Z_L_P_L_: *p* = 0.008, t = 3.69 and Z_L_P_E_: *p* = 0.005, t = 3.86). In contrast, adult females maintained a significantly suppressed respiration rate under combined elevated *p*CO_2_ compared to all other treatments (pairwise-test: Z_L_P_L_: *p =* 0.004, *t* = 3.79, Z_E_P_L_: *p =* 0.007, *t* = 4.22 and Z_L_P_E_: *p =* 0.006, *t* = 4.74).

#### Phytoplankton to zooplankton trophic transfer efficiency

Trophic transfer efficiencies declined in populations exposed to the combined elevated *p*CO_2_ treatment (Z_E_P_E_) compared to the control (Z_L_P_L_). The proportion of carbon ingested that was allocated to growth (i.e., gross growth efficiency [GGE]) declined by 78% (pairwise-test: *p* = 0.007, *t* = 4.35), whilst the proportion of carbon incorporated into growth in relation to the total carbon assimilated (i.e., net growth efficiency [NGE]) declined by 52% (pairwise-test: *p* = 0.012, *t* = 1.91, S3 Table). Significant correlations were found between the multivariate biochemical stoichiometry of the prey to that of the predators (Mantel test: *p* = 0.001, Rho = 0.68). Multiple stepwise search analyses between two trophic levels indicated that the lipid: protein ratio in *C*. *muelleri*, the lipid: carbohydrate and protein: carbohydrate ratio in *I*.*galbana* had the greatest influence on *Acartia tonsa’s* biochemical composition across the 4 treatments (*p* = 0.02, rho = 0.544).

#### Population fecundity success

Different exposure pathways influenced different aspects of the reproductive processes. Egg production rates (EPR) and nauplii recruitment (NR) declined upon indirect elevated *p*CO_2_ exposure compared to ambient conditions (pairwise-test: EPR: *p =* 0.009, *t =* 5.58; NR: *p =* 0.011, *t =* 2.54; Fig 3B). In contrast, egg size decreased through direct elevated *p*CO_2_ exposure (pairwise-test: *p* = 0.009, *t =* 5.91). The combined OA treatment led to an adverse synergistic effect on the EPR and NR, declining both production and recruitment by > 75% (pairwise test, both *p* < 0.001).

## 4.Discussion

This study is the first to directly demonstrate the consequences of elevated *p*CO_2_ on the trophic transfer between copepods and multiple phytoplankton prey species. The subtlety of the processes that affect prey selection and ingestion, and directly and indirectly then affect growth and reproduction of the consumer are shown to be associated with changes in the biochemical stoichiometry of the prey. While biochemical stoichiometry has been implicated before as an important factor modulating the well-known elemental-level ecological stoichiometry [33,34], here for the first time the event is explicitly demonstrated and also associated with ocean acidification (OA).

The impacts of OA on the elemental stoichiometry of phytoplankton have previously been shown to be species-specific. While some species demonstrate no effects under elevated *p*CO_2_ conditions [16,35], other species [10,15,36,37,38] have developed increased C:(N:P) ratios under these conditions. Such deviations have also been seen in mixed-assemblages [12] and phytoplankton communities [39]. In this present study, *C*. *muelleri*, *I*.*galbana* and *T*. *suecica* displayed an insignificant increase C: N under elevated *p*CO_2_. Further, no differences were found in the growth rates or cell size of any of the phytoplankton species between the treatments in our study. The species-specific response to elevated *p*CO_2_ only becomes evident in the phytoplankton’s biochemical composition (Fig 1A and 1B), with *C*. *muelleri* and *I*. *galbana* expressing relative declines in the lipid: protein (*C*.*muelleri and I*.*galbana*), lipid: carbohydrate and protein: carbohydrate (*I*. *galbana*). These differences found across the biochemical composition of *C*. *muelleri* and *I*. *galbana* highlights the importance of not relying solely upon the use of elemental stoichiometry as an indicator of prey quality. This, then, explains how subtle differences in elemental stoichiometry can have important non-linear effects on predation [33] with serious impacts on predator-prey dynamics ranging from a collapse in growth potential [40] to the rejection of prey consumption and a switch in alternative prey (including cannibalism; [41]).

The influence of direct, indirect or combined elevated *p*CO_2_ exposure on the behaviour of copepods is poorly understood. Within this current study, behaviour was assessed through examining prey preference within a mixed prey assemblage that had been reared either under elevated or low CO_2_ levels (P_E_ vs P_L_). Optimum prey size theories for copepods [42,43,44] indicate that *A*. *tonsa* males and females should actively select *T*. *suecica* over *C*. *muelleri* and *I*. *galbana* (calculated using the cell volume of the three prey three species). However, the diatom *C*. *muelleri* was ingested and preferentially selected for at a significantly greater rate compared to the other species when it was grown under elevated *p*CO_2_ levels (P_E_) compared to low *p*CO_2_ levels (P_L_), irrespective of the predator’s own *p*CO_2_ exposure (i.e., Z_E_P_E_ vs Z_L_P_E_). This active selection of elevated *p*CO_2_ reared *C*. *muelleri* suggests that the diatom was a more attractive prey type to the predators in comparison to the other prey reared under elevated *p*CO_2_. Whilst the exact nature of the link between the prey’s biochemical content and predator preference remains unknown, the potential cause-and-effect has clear and important tropho-dynamic implications for life under OA. Here, we see that the pivotal significant difference between the growth and reproduction of copepods reared under elevated *p*CO_2_ was attributable to the biochemical stoichiometry of the prey. Potentially, this could suggest that bottom-up indirect impacts of OA on copepod populations are dependent on the species-specific response of the available prey within the predator’s habitat. Such assumptions would also explain the reported declines in copepod reproduction through bottom-up effects of elevated *p*CO_2_ when predators were fed on a sole prey diet [17], whilst the population structure remained unaffected when individuals were fed on a variety of prey from their natural planktonic communities [45].

Recently there has been a significant rise in research exploring the direct acute, chronic and transgenerational effects of elevated *p*CO_2_ on copepod mortality rates [4], vital rates [8], developmental rates [5,46] and elemental composition [47, 48, 49]. However, little is known of the indirect effects of elevated *p*CO_2_ on copepod population dynamics [9], or indeed of the more natural scenario which incorporates the combined interacting effects of direct and indirect exposure to elevated *p*CO_2_. In this current study, the indirect effects of elevated *p*CO_2_ (i.e., P_E_) predominately influenced the reproduction of *A*. *tonsa*, while the direct exposure (i.e., Z_E_) primarily affected the male copepods respiratory rates. Combining the two exposures (Z_E_P_E_) resulted in adverse synergistic effects to both the fecundity success and respiratory rates of adult females, and the decline across both net and gross growth efficiencies (NGE, GGE, respectively; S3 Table). As the direct effects of elevated *p*CO_2_ on the prey species only affected the biochemical properties (rather than the gross elemental content) of *I*. *galbana* and *C*. *muelleri*, it seems probable that these subtle alterations were the cause of the indirect effects to the copepods reproduction. This observed sensitivity is consistent with our earlier observation [40] showing that rather minor changes in elemental stoichiometry could have a catastrophic impact upon copepod growth even though ingestion rates remained high. In that earlier study the quality of the prey was affected by nutrient stress (N-limitation); here the impact was not through N-starvation but through the more ready availability of the substrate for C-fixation (i.e., CO_2_ (aq)).

While the details of the changes in macromolecule functional groups within *C*. *muelleri* and *I*. *galbana* in response to growth with elevated *p*CO_2_ await further investigation, declines in the FTIR absorption spectra implicate significant changes in lipid: protein and lipid: carbohydrate ratios (Fig 1A and 1B). Both lipid and proteins play critical roles in the somatic growth and reproduction of marine copepods [50]. As *Acartia* lack the biosynthetic capacity for *de novo* synthesis of certain sterols and fatty acids they rely on their dietary intake to meet their metabolic requirements [51]. The different reproductive processes (e.g., gonad development, oogenesis and vitellogenesis) are also energetically expensive and require multiple nutritional components across the different reproductive stages [52]. In *A*. *tonsa* the concentrations of available sterols, fatty acids (e.g., 20:5n3, 22:6n3 and 18:0) and proteins positively correlate to their egg production rate (EPR [51,52]). In contrast, the nutritional requirements for the success of egg hatching in *Acartia* appear to be less specific, with a wide range of fatty acids and sterols proving adequate for egg viability [52]. Together, these likely explain the declines found in the production rates and size of eggs produced under indirect elevated *p*CO_2_ exposure, but with no effects found on the hatching success rates in females.

Coupled with the 75% decrease in population recruitment found under the combined elevated *p*CO_2_ treatment was the 50% decline found in female respiratory rates (Fig 3). Maintaining internal homeostasis under hypercapnia can cause costly energetic trade-offs, due to less energy being allocated to other physiological activities [53]. If respiratory acidosis cannot be compensated for under elevated *p*CO_2_ conditions then organisms can undergo metabolic suppression, which acts as a short-term solution to the acid-base imbalance [54]. However, when this metabolic suppression strategy is adopted for a chronic duration it adversely affect organism fitness through the active repression of critical physiological processes (e.g., protein synthesis), which can decrease an individual’s ability to grow and reproduce [55]. Prior to entering metabolic suppression, though, the energetic cost for an individual in maintaining internal homeostasis under hypercapnia can be alleviated through consumption of increased food quality and/or quantity [56]. Within our study, total prey ingestion rates by females did not alter between the four treatments. Thus the variation in prey quality between the treatments (P_E_ vs P_L_) could explain the lack of respiratory and reproductive impacts in females directly exposed to elevated *p*CO_2_ whilst fed prey reared under ambient conditions (i.e., Z_E_P_L_).

Deviations in an individual’s metabolic rate upon exposure to an environmental perturbation can provide valuable insight into an organism’s ability to preserve internal homeostasis, sustain life history traits and maintain fitness [57]. Research into the metabolic rates of copepods exposed to OA scenarios has emphasised their species-specific response to climate change. Upregulated respiratory rates have been associated with the acute exposure to extreme *p*CO_2_ concentrations (3000 μatm) in adult *Centropages tenuiremis* [58], in addition to the transgenerational exposure of C_V_ *Calanus finmarchicus* (1080–3080 μatm *p*CO_2_, [46]) and *Pseudocalanus acuspes* (900 μatm *p*CO_2_, [7]). However, no change in respiratory costs were linked to the elevated acute exposure (824 μatm *p*CO_2_) of *Acartia clausi* [59] or the high chronic exposure (3000 μatm *p*CO_2_) of C_V_ *C*. *hyperboreus* and C_V_ *C*. *glacialis* [49]. One of the novel aspects of this current study is that it demonstrates the contrasting ontogenic respiratory responses to elevated *p*CO_2_. Whilst adult females suppressed their respiratory rates when exposed to the combined treatment, adult males maintained increased oxygen consumption rates under direct elevated *p*CO_2_ exposure, regardless of the status of the prey they ingested (Z_E_P_L_ and Z_E_P_E_; Fig 3A).

Further studies are required to investigate how the effects found in this study relate to trophic interactions between wild populations. However, there is every reason to expect the core observations to match, because of the commonality of stoichiometric ecology as a driver in all systems. Thus, trophic dynamics within the plankton food webs are subject to potential feedback loops associated with nutrient regeneration; consumption of good quality prey results in high regeneration rates of nutrients, which maintains the good quality status [34]. Under OA there is scope for additional feedback events. Thus, phytoplankton growth under elevated *p*CO_2_ generates different scales of basification (increase in pH with C-fixation), which is expected to affect plankton succession [54]. From the current study, we can see scope for an additional level of factors affecting phytoplankton selectivity that may develop through the discriminatory activity of the grazers driven by changes in prey’s biochemical stoichiometry. The totality of these interactions will take some additional effort to fully understand, but for now the combined implications of the results from this study, coupled with that of the phytoplankton-only study of Flynn *et al* [60] gives us clear cause to suspect that secondary production mediated by copepods has the potential to alter significantly under OA.

## Supporting Information

S1 Table Detailed methodology. (DOCX)

S2 Table Seawater carbonate chemistry. (DOCX)

S3 Table Trophic transfer efficiencies of adult female *Acartia tonsa*. (DOCX)

S1 Text Detailed methodology. (DOCX)
