# Supplementary material for: Ocean Acidification Affects the Phyto-Zoo Plankton Trophic Transfer Efficiency
Source: PLoS One. 2016 Apr 15;11(4):e0151739. doi: 10.1371/journal.pone.0151739 (PMC4833293; doi:10.1371/journal.pone.0151739)
Supplement: S1 Table — (DOCX) [file pone.0151739.s001.docx]

$\hat{\alpha}_{i}=\frac{{{\ln((r}_{i}-n}_{i})/n_{io})}{\sum_{j=1}^{m} ln \left( \frac{n_{jo}-r_{j}}{n_{jo}} \right)}, i=1, \ldots., m$ $X\sigma= ab\sqrt{\frac{\sigma_{a}}{a}+\frac{\sigma_{b}}{b}}+\ldots$**Table legend**

**S1 Table.** Specific infrared spectral peaks and assigned functional groups used for microalgae (*Isochrysis galbana*, *Tetraselmis suecica* and *Chaetoceros muelleri*) and copepods (*Acartia tonsa*) during quantification of biochemical of stoichiometry. Assigned functional groups for lipids and proteins were chosen to represent the biochemical groups due to their direct correlations with absolute values [7,9], and the carbohydrate range used to incorporate both polysaccharides and cellulose components [16]. ^†^ denotes the carbohydrate wavelength used for the copepods within this study, and represents the carbohydrate backbone within crustaceans [17]. Peak absorbance within each functional group was used to calculate the relative biochemical stoichiometry between the biochemical groups (method as described in [16]). *v =* symmetric stretch, *v*_as_ asymmetric stretch.

| **Biochemical group** | **Assigned functional group** | **Wavelength** |
| --- | --- | --- |
|  |  |  |
| Carbohydrate | *v*C—O —C/*vas* P= O, *v* C—O , polysaccharides and cellulose | 1200-950  (1040-1070) ^†^ |
|  |  |  |
| Lipids | *v*C=O, membrane lipids and fatty acids | 1750-1730 |
|  |  |  |
| Protein | *v*C=O, amide I | 1665-1625 |
|  |  |  |
|  |  |  |

**S1 Table**
