# Supplementary material for: Ocean Acidification Affects the Phyto-Zoo Plankton Trophic Transfer Efficiency
Source: PLoS One. 2016 Apr 15;11(4):e0151739. doi: 10.1371/journal.pone.0151739 (PMC4833293; doi:10.1371/journal.pone.0151739)
Supplement: S2 Table — (DOCX) [file pone.0151739.s002.docx]

# Supplementary Information S2 Table

# Seawater carbonate chemistry

## Table legend

**S2 Table.** Seawater carbonate chemistry (average ± 1 SE) of the phytoplankton and copepod cultures during the experiment. Phytoplankton data averaged across the light and dark photoperiod. † denotes that *p*CO_2_ was calculated through alkalinity (A_T_) and pH. P_L_: plankton reared under ambient *p*CO_2_ levels, P_E_: plankton reared under elevated *p*CO_2_ levels, Z_L_P_L_: both plankton prey and copepod predators reared under ambient *p*CO_2_ levels, Z_E_P_L_: prey reared under ambient *p*CO_2_ levels and predators reared under elevated levels, Z_L_P_E_: prey reared under elevated *p*CO_2_ levels and predators reared under ambient levels, and Z_E_P_E_: both prey and predator reared under elevated *p*CO_2_ levels.

**S2 Table**

|  |  |  |  |  |  |  |  |  |  |  |  |
| --- | --- | --- | --- | --- | --- | --- | --- | --- | --- | --- | --- |
| **Species** | | **Treatment** | **Temperature** (ºC) |  | **Salinity** |  | **A_T_**  (µmol kg^-1^) |  | **pH**  (NBS) |  | ***p*CO_2_**^†^  (µatm) |
|  |  |  |  |  |  |  |  |  |  |  |  |
| **Prey** | *Chaetoceros muelleri* | P_L_ | 19.8 ± 0.17 |  | 27.1 ± 0.36 | | 2506 ± 55.63 |  | 8.308 ± 0.015 |  | 349 ± 39.0 |
|  |  | P_E_ | 20.8 ± 0.14 |  | 26.3 ± 0.10 | | 2529 ± 16.36 |  | 7.850 ± 0.005 |  | 1174 ± 51.10 |
|  |  |  |  |  |  |  |  |  |  |  |  |
|  | *Isochrysis*  *galbana* | P_L_ | 20.4 ± 0.09 |  | 26.7 ± 0.05 | | 2496 ± 22.99 |  | 8.230 ± 0.006 |  | 420 ± 18.04 |
|  |  | P_E_ | 20.7 ± 0.14 |  | 27.0 ± 0.08 | | 2499 ± 30.37 |  | 7.911 ± 0.015 |  | 1002 ± 97.50 |
|  |  |  |  |  |  |  |  |  |  |  |  |
|  | *Tetraselmis*  *suecica* | P_L_ | 20.1 ± 0.07 |  | 27.3 ± 0.10 | | 2476 ± 10.39 |  | 8.331 ± 0.017 |  | 345 ± 42.25 |
|  |  | P_E_ | 20.2 ± 0.08 |  | 27.3 ± 0.09 | | 2549 ± 14.87 |  | 7.896 ± 0.016 |  | 1059 ± 42.25 |
|  |  |  |  |  |  |  |  |  |  |  |  |
|  |  |  |  |  |  |  |  |  |  |  |  |
|  |  |  |  |  |  |  |  |  |  |  |  |
| **Predator** | *Acartia tonsa* | Z_L_P_L_ | 20.4 ± 0.13 |  | 26.7 ± 0.06 | | 2403 ± 06.08 |  | 8.212 ± 0.016 |  | 429 ± 23.40 |
|  |  | Z_E_P_L_ | 20.3 ± 0.18 |  | 26.9 ± 0.06 | | 2431 ± 25.87 |  | 7.719 ± 0.017 |  | 1210 ± 68.30 |
|  |  | Z_L_P_E_ | 21.0 ± 0.06 |  | 26.9 ± 0.08 | | 2433 ± 10.77 |  | 8.187 ± 0.014 |  | 462 ± 20.90 |
|  |  | Z_E_P_E_ | 20.7 ± 0.12 |  | 26.6 ± 0.16 | | 2450 ± 25.80 |  | 7.714 ± 0.017 |  | 1217 ± 81.20 |
|  |  |  |  |  |  | |  |  |  |  |  |
|  |  |  |  |  |  |  |  |  |  |  |  |
