# Supplementary material for: Ocean Acidification Affects the Phyto-Zoo Plankton Trophic Transfer Efficiency
Source: PLoS One. 2016 Apr 15;11(4):e0151739. doi: 10.1371/journal.pone.0151739 (PMC4833293; doi:10.1371/journal.pone.0151739)
Supplement: S3 Table — (DOCX) [file pone.0151739.s003.docx]

# Supplementary Information S3 Table

#

# Trophic transfer efficiencies of adult female *Acartia tonsa*

## Table legend

**S3 Table.** Female body weight (F, µg C), and weight-specific (gC gC^-1^ day^-1^) ingestion (I), respiration (R) and production (P) rates of female *A.tonsa* exposed to elevated *p*CO_2_ directly(Z_E_P_L_), indirectly(Z_L_P_E_) and a combination of both (Z_E_P_E_). These metabolic rates were used to determine the average growth gross efficiency (GGE) and net growth efficiency (NGE) of the population. Values are averages and calculated standard deviations (± 1SD) using error propagation. Bold values denote significance from ambient populations (Z_L_P_L_).

**S3 Table.**

|  |  |  |  |  |  |  |  |  |  |  |  |
| --- | --- | --- | --- | --- | --- | --- | --- | --- | --- | --- | --- |
| **Treatment** | **F** |  | **I** |  | **R** |  | **P** |  | **GGE** |  | **NGE** |
| **Z_L_P_L_** | 4.129 ± 0.15 | | 1.675 ± 0.43 | | 0.181 ± 0.04 | | 0.198 ± 0.09 |  | 0.120 ± 0.06 |  | 0.518 ± 0.27 |
|  |  |  |  |  |  |  |  |  |  |  |  |
| **Z_E_P_L_** | 4.821 ± 0.33 | | 1.424 ± 0.67 | | 0.185 ± 0.06 | | **0.093 ± 0.08** |  | 0.065 ± 0.06 |  | 0.334 ± 0.30 |
|  |  |  |  |  |  |  |  |  |  |  |  |
| **Z_L_P_E_** | 4.356 ± 0.06 | | 1.283 ± 0.47 | | 0.197 ± 0.04 | | **0.108 ± 0.03** |  | 0.084 ± 0.04 |  | 0.353 ± 0.12 |
|  |  |  |  |  |  |  |  |  |  |  |  |
| **Z_E_P_E_** | 4.591 ± 0.12 | | 1.468 ± 0.45 | | **0.087 ± 0.03** | | **0.038 ± 0.02** |  | **0.026 ± 0.02** |  | **0.301 ± 0.21** |
|  |  |  |  |  |  |  |  |  |  |  |  |
