# Supplementary material for: Ocean Acidification Affects the Phyto-Zoo Plankton Trophic Transfer Efficiency
Source: PLoS One. 2016 Apr 15;11(4):e0151739. doi: 10.1371/journal.pone.0151739 (PMC4833293; doi:10.1371/journal.pone.0151739)
Supplement: S1 Text — (DOCX) [file pone.0151739.s004.docx]

**S1 Text**

**Detailed Methodology**

# Method

Throughout the following, subscript L and subscript E refer to treatments as low (ambient) or elevated (OA) *p*CO_2_ respectively, as applied to zooplankton (i.e., Z_L_, Z_E_) or phytoplankton (i.e., P_L_, P_E_). Direct treatments are thus indicated as Z_E_P_L_, indirect as Z_L_P_E_, and combined as Z_E_P_E_, with the control as Z_L_P_L_.

## 1.1. Cultures

The calanoid copepod, *Acartia tonsa*, was obtained originally from the Environment and Resource Technology (ERT), Orkney, UK. Stock cultures were maintained at 24.5°C (± 0.54) with a 14:10 photoperiod (4- 9 μmol photons m^-2^ s^-1^) in aerated (392 ± 27 µatm *p*CO_2_; nominally 400 µatm) filtered (0.22µm) seawater. These stock cultures of *A.tonsa* were fed a mixed prey diet of the prymnesiophyte *Isochrysis galbana* (Strain CCAP 927/ 1), prasinophyte *Tetraselmis suecica* (Strain CCAP 66/ 22C) and diatom *Chaetoceros muelleri* (Strain CCAP 1010/ 3). The microalgae were grown separately in nutrient replete seawater-based medium (88.2 and 5.5 µmol L^-1^ for NO_3_^-^ and PO_4_^3-^ respectively; mole N:P ratio 16:1), at 22 ± 1.8 °C in a 18:6 hour light: dark cycle (cool white fluorescent tubes at 50 μmol photons m^-2^ s^-1^). The algae were fed to the copepods in a ratio of 1:1:1 relative to the carbon biomass concentration of the algae (total carbon biomass added = 1 µg C mL^-1^).

## 1.2. Carbonate manipulation

OA is a long-term event, integrating over space and time. In experiments, typically attempts are made to mimic the simplest chemico-physico effect of OA by exposing organisms to elevated partial pressures of carbon dioxide (*p*CO_2_) in keeping with those projections expected under different emission scenarios. Thus, while often direct reference is made in the literature to the influence of OA, in reality reference should more appropriately be made to the influence of elevated *p*CO_2_ that aligns with OA. We henceforth use *p*CO_2_ as a proxy to represent OA scenarios. Both trophic levels (*A.tonsa* and the three prey species: *I. galbana, T.suecica* and *C.muelleri*) were exposed to two *p*CO_2_ scenarios; (i) low: present-day *p*CO_2_ concentrations of 400 µatm, and (ii) elevated: worst-case scenario for the year 2100, 1000 µatm (RCP 8.5 [1]). These two *p*CO_2_-climate scenarios were achieved through saturation of seawater through the aeration of air:CO_2_ mixture attained using Mass Flow Controllers (Aalborg GFC17). Measurements of pH were made through a three-point decimal place Omega PHB-121 bench top microprocessor pH meter cross-referenced with a WTW 315i portable meter (2A10-101T), both calibrated with pH 7.01 & 10.01 (NBS scale). Total alkalinity (A_T_, measured by open cell pentiometric titration using an AS-ALK2 Gran Titrator, Apollo SciTech, USA), pH, salinity and temperature were used to calculate the seawater *p*CO_2_ (µatm) through the programme CO2 SYS [2], using the K_1_, K_2_ constants from Mehrbrach *et al* [3], as refitted by Dickson and Millero [4].

## 1.3. Experimental design

### 1.3.1. Phytoplankton

All species were cultured separately in semi-continuous cycles (effective dilution rate: *T. suecica* 0.30 d^-1^, *I. galbana* and *C. muelleri*: 0.35 d^-1^). Duplicate cultures of each species were used for both *p*CO_2_ treatments (500 mL, total n = 18). *I. galbana*, *C. muelleri* and *T. suecica* cultures (450 mL) were aerated with air at the required *p*CO_2_ concentration (either low or elevated) through a sterilised glass airline via an air-filter (0.2µm) at a rate of ca. 52 mL min^-1^. All phytoplankton cultures were started with low cell densities (*I. galbana* 50,000 cell mL^-1^, *T. suecica* 5,000 cell mL^-1^ and *C. muelleri* 25,000 cells mL^-1^) in order to decrease the influence of algal metabolic processes on the carbonate chemistry of the bulk seawater. The cultures were started in media of the required *p*CO_2_, and aeration was initiated 24-hours after the experiment started to promote growth of the low cell density culture [5]. Temperature, salinity and pH were measured daily at the beginning and end of the photoperiod, whilst A_T_ was measured every 48 hours. All cultures were exposed to the nominal *p*CO_2_ treatment for at least 12 generations.

Cell number (cells mL^-1^), size (as equivalent spherical diameter, µm) and biovolume (µm^3^ mL^-1^) across all replicates were analysed at the end of each light cycle using a Multisizer 4 Coulter Counter (Beckman, USA). Every 48hrs, at the semi-continuous exchange point, cells were collected from each culture for elemental stoichiometry and biochemical analysis. Cellular carbon (µg C mL^-1^), nitrogen (µg N mL^-1^) and the C: N of each species from both *p*CO_2_ concentrations were analysed using an elemental analyser coupled with an isotope ratio mass spectrometer (SerCon GSL) using isoleucine as the standard. Biochemical stoichiometry of each species cultured at different *p*CO_2_ concentrations was assessed through Fourier Transform Infrared (FTIR) spectroscopy (PerkinElmer Spectrum 2) over a range of 450- 4000 cm^-1^ and at a resolution of 4 cm^-1^ (method as described in [6]). The absorbance of infra-red at the assigned spectral wave length is proportional to the concentration of the corresponding bond or molecule within the sample, allowing quantitative analysis of the different functional groups [7,8,9]. Here, a semi-quantitative approach was adopted and integrated FTIR band ratios of specific spectral areas were used to assess the relative difference between biochemical stoichiometries (outlined in S1 Table); (i) lipids: protein, (ii) protein: carbohydrate, and (iii) carbohydrate: lipid. All spectra bands were verified with standards; bovine serum albumin (BSA, grade > 98 %, Sigma-Aldrich) for protein, glucose (grade > 99.5 %, Sigma-Aldrich) for carbohydrates and glycerol tripalmitate (grade > 99%, Sigma-Aldrich) for lipids. FTIR spectra were baseline corrected and normalised to the silicate peak (1074 cm^-1^) for *C. muelleri* and the amide II bond peak (maximum ordinate values over 1520- 1565 cm^-1^) for *I. galbana, T. suecica* and *Acartia tonsa* to minimise interference between replicate samples.

### 1.3.2. Copepods

The two *p*CO_2_ concentrations (nominally 400 and 1000 µatm; termed “low” and “elevated” from here on) were combined in a matrix between the two trophic levels to produce 4 treatments: (i) Z_L_P_L_: zooplankton (*A.* *tonsa*) reared under low *p*CO_2_ levels fed mixed phytoplankton (*I.* *galbana, C. muelleri* and *T. suecica*) also reared under low *p*CO_2_ levels, (ii) Z_L_P_E_: zooplankton reared under low *p*CO_2_ levels fed mixed phytoplankton reared under elevated (RCP 8.5) *p*CO_2_ levels, (iii) Z_E_P_L_: zooplankton reared under elevated *p*CO_2_ levels fed mixed phytoplankton reared under low *p*CO_2_ levels, (iv) Z_E_P_E_: zooplankton reared under elevated *p*CO_2_ levels fed mixed phytoplankton also reared under elevated *p*CO_2_ level. The copepod experiment did not commence until all prey species had been exposed to the appropriate *p*CO_2_ treatment (P_L_ or P_E_) for at least 8 generations.

The copepods were exposed to these four treatments for an entire life-cycle, from generation 1(G_1_) early nauplii stages (N_I_) through to G_2_ mid-late nauplii stages (N_III-IV_). The N_I_ used to initiate the experiment were produced from 3 separate culturing tanks (800L), each with > 20,000 mature adults. N_I_ hatchlings (≤ 12 hours from hatch and ≤ 4 hours age difference between individuals) were located under a microscope (Nikon SMZ800) and pipetted into autoclaved 1 L Schott flasks (4 replicates per treatment, density 890 ind^-1^ L^-1^) filled with the allocated *p*CO_2_ concentration and prey diet. Each bottle was maintained on a rotating plankton wheel at 2 rpm in a constant temperature room at 21 ºC with a 14:10 light: dark photoperiod (4- 9 µmol m^-2^ s^-1^). Water exchange (90 %) occurred every 48 hours at the end of the copepod light cycle, an event synchronised with the semi-continuous water exchange of the prey species. The frequent copepod water exchange minimised the seawater carbonate drift and ensured saturating prey quantities (> 1 µg C mL^-1^) for the copepods. During the water exchange temperature, salinity, pH and A_T_ were measured across all replicates, and *p*CO_2_ concentrations calculated as described above. Vital rates, behaviour and trophic transfer efficiencies were calculated in adult copepods after one generation of exposure.

***Vital rates*:** when > 50% of the *Acartia* population had reached maturity in the preceding water exchange then fecundity success, respiration rates and ingestion rates were measured in adult males and females across the four treatments. For fecundity success, 5- 8 females from each replicate population (n = 20- 32 individuals per treatment) with an attached spermatophore were removed and placed individually into 30 mL vials filled with their assigned treatment and saturating prey quantities of their allocated prey (>1µg C mL^-1^). Each vial was pre-lined with a 150 μm nylon mesh bottom to separate eggs from the female to prevent egg cannibalism. Females were held for 24- 30 hours to lay eggs. Egg production rates (EPR [eggs female^-1^ day^-1^]), egg hatching success (EHS [%]) and nauplii recruitment (NR [nauplii female^-1^ day^-1^]) across the four treatments were calculated as described in Cripps *et al* [10].

Ingestion rates (µg C ind^-1^ day^-1^) of adult males and females were measured separately. A sufficient number of copepods (males: 250 ind^-1^ L^-1^, females:167 ind^-1^ L^-1^) were transferred from the experimental population replicates to 60 mL tissue culture flasks (6-8 replicates per life stage for each treatment) and filled with filtered (0.2 µm) sterilised seawater of the required *p*CO_2_ concentration. Prey (*I*. *galbana, C. muelleri* and *T. suecica*), reared under low or elevated *p*CO_2_, were added to the corresponding predator tissue culture flasks at the same concentration as used for the stock populations; initial (t_1_) cell counts were taken. Flasks were placed on the plankton wheel (2 rpm) in a constant temperature room (23.9 ± 0.63 ºC) in the dark for > 24 hours to capture any diel feeding patterns. At the end of the experiment, each flask was gently inverted for one minute before t_2_ sub-sample (3 x 5 mL) was measured. Copepods were filtered out onto 60 µm nylon mesh, re-suspended in 10 mL of filtered seawater and enumerated under the microscope and scored as live or dead. Ingestion rates were calculated through Frost’s [11] equations.

Oxygen consumption rates were measured across 2 different stages of *A.tonsa* (G_1_: male and female) using an optical fluorescence- based oxygen respirometry (Fibox 3 LCD trace transmitter, PreSens, Germany). A planar oxygen sensor spot (diameter of 5mm with optical isolation: type PSt3; PreSens, Germany) was secured (silicon glue) to the bottom of each of 50 cylindrical glass vials (2.62 mL). The sensor spots consist of an oxygen-sensitive foil with an immobilised fluorescent dye that undergoes dynamic fluorescence quenching in the presence of oxygen. All sensor spots were calibrated with 2% sodium sulphite and 100% oxygen saturated filtered (0.2µm) seawater (at 20^o^C and 28.8 salinity) prior to the experiments. The routine respiratory rates of adult males and females were measured separately and at different time intervals. The required number of copepods for each measured life stage (averages of female: 2.31 ind^-1^ mL^-1^ and males: 3.85 ind^-1^ mL^-1^) were transferred into the vials using a sterile glass pipette (8-10 replicates). Each vial was filled to the top with seawater of the appropriate *p*CO_2_, sealed with silicone rubber bungs and wrapped in parafilm to prevent gaseous exchange during the experiments. For each *p*CO_2_ treatment 2 duplicate controls (filtered seawater) were used to account for any background changes due to temperature fluctuation and/or oxygen production by bacteria (any background changes were subtracted from the experimental vials prior to calculating respiratory rates). The vials were placed into a temperature bath that matched the temperature of the constant temperature room. The *in-situ* temperature was monitored continuously through a temperature sensor probe (*PT*_1000_, PreSens). Oxygen consumption rates were recorded every hour for a 6-8 hour period. Measurements over the first hour were excluded from the analyses to account for acclimation of the individuals within the vials. At the end of the experimental duration, individuals were enumerated and subsequently fixed with 5 % iodine for size analyses (prosome length), which was carried out immediately. The oxygen consumption of the individuals within the vials was calculated as the linear part of the time-dependent oxygen concentration curves through linear regression (*f = y0+a.x*).

***Behaviour*:** Adult male and female prey preference under direct, indirect and combined exposure to elevated *p*CO_2_, was calculated using Chesson’s prey selection index [12].

$\hat{\alpha}_{i}=\frac{{{\ln((r}_{i}-n}_{i})/n_{io})}{\sum_{j=1}^{m} ln \left( \frac{n_{jo}-r_{j}}{n_{jo}} \right)}, i=1, \ldots., m$ Eq.1

Here, $\alpha$_i_ is the Chesson prey selection index, $n_{i0}$ is the number of prey items of prey type *i* present at the beginning of the experiment, $r_{i}$ is the number of prey type *i* (cell number) consumed by each predator (adult males or females), and *j* is the number of different prey types (*C. muelleri* j = 1*, I. galbana* j = 2*, T. suecica j = 3*). In this study *m*= 3 different prey species available. The Chesson prey selection index ranges from 0 (complete avoidance) to 1 (sole selection of that prey type). Thus here, with 3 prey types, an $\alpha$_i_ close to 1/*m* (0.333) represents random feeding, a value greater than 1/m (> 0.333) indicates positive selection (hereafter referred to as active selection) and less than 1/m (< 0.333) as negative selection (hereafter referred to as avoidance).

***Copepod chemical stoichiometry:*** At the end of G_1_, adult males and females (between 1-5 days old) were collected for elemental stoichiometry (µg C ind^-1^, µg N ind^-1^ and C:N) and biochemical composition across the four treatments. The carbon and nitrogen content of the adults were measured separately for males (8-10 replicates per treatment, 15-25 individuals per replicate) and females (8-10 replicates per treatment, 10-15 individuals per replicate). Individuals were placed into tin cups (6x4 mm; Exeter Analytical, UK), immediately frozen and stored -80 ^o^C until analysis. The relative difference between the biochemical compositions of *A. tonsa* adults were assessed at the same time interval as the elemental stoichiometry. Adults were washed with distilled water on a 200µm mesh to remove the salt. Individuals were transferred into 1.5 mL micro centrifuge tubes, frozen at -80 ^o^C, freeze dried (< 24 hours after being frozen) and then homogenised prior to FTIR analysis. For this, the exact same method and quantification for biochemical stoichiometric analysis as used for the prey was employed for the predators.

***Trophic transfer efficiency:*** the influence of different *p*CO_2_ treatments (direct, indirect and combined) on the trophic transfer efficiency was assessed through calculating the carbon allocation budgets of adult females in G_1_. All measured metabolic rates were converted into carbon equivalents; ingestion rates (I, gC gC^-1^ d^-1^), EPR were used as an index for female growth (G, gC gC^-1^ d^-1^); respiration rates (nL O_2_ ind^-1^ min^-1^) were converted into respiratory carbon equivalents (R, gC gC^-1^ d^-1^) using the respiratory quotient of 0.97 [13, 14]. The proportion of carbon ingested (I) that was allocated to growth (G) was calculated as Gross Growth Efficiency (GGE = G/I). The proportion of carbon incorporated into growth in relation to the total carbon assimilated was calculated as Net Growth Efficiency (NGE = G/ G+R). The standard deviation (Xσ) for the calculated transfer efficiencies (NGE and GGE) and weights-specific rates (I, R and G) were calculated through Eq.2 to incorporate error propagation.

$X\sigma= ab\sqrt{\frac{\sigma_{a}}{a}+\frac{\sigma_{b}}{b}}+\ldots$ Eq.2

Here, σ refers to the standard deviation of the individual parameters (a,b: respiration, ingestion, production, growth or body weight) used to calculate the carbon budget or weight specific growth rate.

## 1.4. Statistical analyses

### 1.4.1 Phytoplankton

The influence of *p*CO_2_ on the growth rates (cells mL^-1^ and BV µm^3^ mL^-1^), cell size (µm), carbon content (µg C), nitrogen content (µg N) and C:N ratios of the three phytoplankton species were analysed using permutational multivariate analysis of variance (PERMANOVA). All dependent variables were assembled into a resemblance matrix using Euclidean distance and analysed using a factorial design with two crossed fixed factors; (i) species (*I. galbana, T. suecica* and *C. muelleri*), and (ii) treatment (P_L_ and P_E_). An additional nested factor of time was incorporated into the ‘treatment’ factor for two of the dependent variables (growth rate and cell size). Main effects and pairwise comparisons of the different factors were analysed through unrestricted permutations of raw data. If a low number of permutations was generated then the *p*-value was obtained through random sampling of the asymptotic permutation distribution, using Monte Carlo tests. For each dependent variable the dispersion across the factors was first analysed using permutational dispersion. Because cell size had a significantly different dispersion across the different *p*CO_2_ levels (both, *p* = < 0.05), cell size was transformed (log (χ+ 1)) prior to the PERMANOVA analysis. Fixed factor (P_L_ and P_E_) multivariate analysis (PERMANOVA) was used to compared the combined biochemical stoichiometry between the treatments for each species, followed by a one-way fixed factor analysis of variance to compare each stoichiometric ratio between the 2 *p*CO_2_ treatments (P_L_ and P_E_). The lipid: carbohydrate and carbohydrate: protein ratios in *I. galbana* were transformed prior to analysis as each ratio had a significantly different dispersion across the different *p*CO_2_ levels (*p* = < 0.05). An α-level of *p* = ≤ 0.05 was used for assessing statistical significance. Analyses were carried out in PRIMER-e (version 6.1.15) with the PERMANOVA add-on (version 1.0.3, Plymouth Marine Laboratory, Plymouth, UK) and R-software (version 3.2.1).

### 1.4.2. Copepods

The influence of direct, indirect and combined elevated *p*CO_2_ exposure on the individual vital rates (fecundity success [EPR: female^-1^ day^-1^, ES: µm^3^, EHS: % and NR: female^-1^ day^-1^], ingestion rates [µg C ind^‑1^ day^-1^] and respiration rates [nL O_2_ ind^-1^ min^-1^]), behaviour (ɑ-index) and elemental stoichiometry (C, N and C:N) of *Acartia tonsa* were analysed using PERMANOVA factorial design with two crossed fixed factors; (i) treatment (Z_L_P_L_, Z_E_P_L_, Z_L_P_E_ and Z_E_P_E_) and (ii) life stage (for respiration and ingestion only). Differences in the copepods relative biochemical compositions between the treatments were analysed using the same method employed for the phytoplankton. Means and calculated standard deviations (see Eq. 2) of trophic transfer efficiencies (GGE and NGE) and weights-specific rates (I, R and G) were compared through a fixed-factor analysis of variance design between the treatments [15]. Correlations between the multivariate biochemical stoichiometric ratios of the prey and the predators were assessed through a Mantel test, using Spearman’s rank correlation coefficient (rho). Multiple stepwise search analyses (BVSTEP) determined which biochemical component across the 3 prey species (lipid: protein, lipid: carbohydrate and protein: carbohydrate of *C. muelleri*, *I. galbana* and *T. suecica* under both P_L_ and P_E_) had the greatest influence on the predator’s composition (lipid: protein, lipid: carbohydrate and protein: carbohydrate of Z_L_P_L_, Z_E_P_L_, Z_L_P_E_ and Z_E_P_E_ populations). The BVSTEP routine successively adds and removes a variable to obtain the optimum correlation between the zooplankton and prey’s composition, using spearman’s correlation coefficient. An α-level of *p* = ≤ 0.05 was used for assessing statistical significance across main tests, and Bonferroni corrections were incorporated during multiple testing between the 4 treatments using an α-level of *p* = ≤ 0.0125.

# References

1. Vuuren DP, Edmonds J, Kainuma M, Riahi K, Thomson A, *et al.* The representative concentration pathways: an overview. Clim change. 2011; 109: 5– 31.
2. Pierrot D, Lewis E, Wallace DWR. CO2sys ms excel program developed for CO_2_ system calculations. Ornl/ cdiac-105. Carbon dioxide information analysis center oak ridge national laboratory us department of energy oak ridge.2006.
3. Mehrbrach C, Culberson CH, Hawley JE, Pytkowicz RM. Measurement of apparent dissociation-constants of carbonic-acid in seawater at atmospheric pressure. Limnol Oceanorgr. 1973; 18: 897- 907.
4. Dickson AG, Millero FJ. comparison of the equilibrium constants for the dissociation of carbonic acid in seawater media. Deep Sea Res*.* 1987; *PT I* 34:1733- 1743.
5. Shi DI, Xu Y, Morel FMM. Effects of pH/pCO_2_ control method in the growth medium of phytoplankton. Biogeosci. 2009; 6:119- 1207.
6. Mayers JJ, Flynn KJ, Shields RJ. Rapid determination of bulk microalgal biochemical composition by fourier-transform infrared spectroscopy. Bioresour Technol. 2013; 148: 215 –20.
7. Giordano M, Kansiz M, Heraud P, Beardall J, Wood B, *et al*. Fourier transform infrared spectroscopy as a novel tool to investigate changes in intracellular macromolecular pools in the marine microalga *Chaetoceros muellerii* (bacillariophyceae). J Phycol*.* 2001; 37: 271–279.
8. Fanesi A, Raven JA, Giordano M. Growth rate affects the responses of the green alga *Tetraselmis suecica* to external perturbations. Plant Cell Environ. 2013; 37: 512– 9.
9. Meng Y, Yao C, Xue S, Yang H. Application of fourier transform infrared (FTIR) spectroscopy in determination of microalgal compositions. Bioresource Technol*.* 2014; 151:347– 54.
10. Cripps G, Lindeque PK, Flynn KJ. Have we been underestimating the effects of ocean acidification in zooplankton? Glob Change Biol*.* 2014; 20: 3377- 3385.
11. Frost BW. Effects of size and concentration of food particles on the feeding behaviour of the marine planktonic copepod *Calanus Pacificus*. Limnol Oceanorgr. 1972; 17: 805-815.
12. Chesson J. The estimation and analysis of preference and its relationship to foraging models. Ecology*.* 1983; 64:1297-1304.
13. Mauchline J. The biology of calanoid copepods. Blaxter JHS, Southward AJ, Tyler PA (eds). Advances in Marine Biology. London. Academic press ltd. 1998.
14. Frangoulis C, Carlotti F, Eisenhauer L, Zervoudaki S. Converting copepod vital rates into units appropriate for biogeochemical models. Prog Oceanogr. 2010; 84: 43– 51.
15. Cohen BH. Calculating a factorial ANOVA from means and Standard deviations. Understanding Statistics. 2002; 1: 191-203.
16. Stehfest K, Toepel J, Wilhelm C. The application of micro-FTIR spectroscopy to analyse nutrient stress-related changes in biomass composition of phytoplankton algae. Plant Physiol Biochem. 2005; 43:717– 26.
17. Prabu K, Natarajan E. Isolation and FTIR spectroscopy characterisation of chitin from local sources. Pelagia Research Library. 2012; 3:1870-1875.
